# Supplementary figures and images for: Biochemical Characterisation of Human Transglutaminase 4
Source: Int J Mol Sci. 2021 Nov 18;22(22):12448. doi: 10.3390/ijms222212448 (PMC8619550; doi:10.3390/ijms222212448)

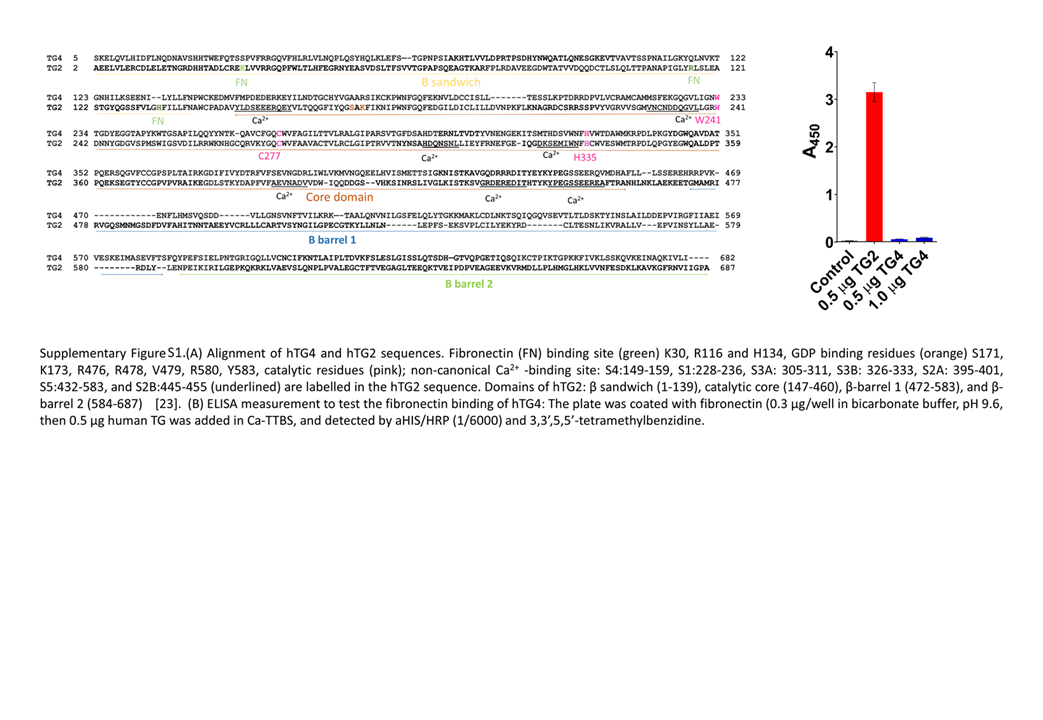

Supplement: Supplementary file 1 [file ijms-22-12448-s001.zip › Supplementary Figure S1.png]
